# Role of RecA and the SOS Response in Thymineless Death in *Escherichia coli*

**DOI:** 10.1371/journal.pgen.1000865

**Published:** 2010-03-05

**Authors:** Natalie C. Fonville, David Bates, P. J. Hastings, Philip C. Hanawalt, Susan M. Rosenberg

**Affiliations:** 1 Department of Molecular and Human Genetics, Baylor College of Medicine, Houston, Texas, United States of America; 2 Interdepartmental Graduate Program in Cellular and Molecular Biology, Baylor College of Medicine, Houston, Texas, United States of America; 3 Department of Molecular Virology and Microbiology, Baylor College of Medicine, Houston, Texas, United States of America; 4 Department of Biological Sciences, Stanford University, Stanford, California, United States of America; 5 Department of Biochemistry and Molecular Biology, Baylor College of Medicine, Houston, Texas, United States of America; 6 Dan L. Duncan Cancer Center, Baylor College of Medicine, Houston, Texas, United States of America; Stanford University School of Medicine, United States of America

## Abstract

Thymineless death (TLD) is a classic and enigmatic phenomenon, documented in bacterial, yeast, and human cells, whereby cells lose viability rapidly when deprived of thymine. Despite its being the essential mode of action of important chemotherapeutic agents, and despite having been studied extensively for decades, the basic mechanisms of TLD have remained elusive. In *Escherichia coli*, several proteins involved in homologous recombination (HR) are required for TLD, however, surprisingly, RecA, the central HR protein and activator of the SOS DNA–damage response was reported not to be. We demonstrate that RecA and the SOS response are required for a substantial fraction of TLD. We show that some of the Rec proteins implicated previously promote TLD via facilitating activation of the SOS response and that, of the roughly 40 proteins upregulated by SOS, SulA, an SOS–inducible inhibitor of cell division, accounts for most or all of how SOS causes TLD. The data imply that much of TLD results from an irreversible cell-cycle checkpoint due to blocked cell division. FISH analyses of the DNA in cells undergoing TLD reveal blocked replication and apparent DNA loss with the region near the replication origin underrepresented initially and the region near the terminus lost later. Models implicating formation of single-strand DNA at blocked replication forks, a SulA-blocked cell cycle, and RecQ/RecJ-catalyzed DNA degradation and HR are discussed. The data predict the importance of DNA damage-response and HR networks to TLD and chemotherapy resistance in humans.

## Author Summary

A long-standing enigma in the fields of DNA repair and cancer chemotherapy is why it is that cells starved of the base thymine die rapidly. This process, called thymineless death (TLD), is conserved in bacterial, yeast, and human cells and is the mode of action of important cancer chemotherapeutic drugs. Tumors that become resistant to those drugs have ceased to die from TLD. Despite its ubiquity, importance, and having been studied for more than 50 years, the mechanism(s) of TLD remained elusive. Here we show that a large fraction of TLD requires RecA, the central protein in homologous recombinational (HR) DNA repair, and activation of the bacterial DNA–damage (or SOS) response, which RecA controls. We find that of the 40 or so proteins upregulated during an SOS response, SulA, an inhibitor of cell division, accounts for most of how SOS–activation causes TLD. In cells undergoing TLD, we observe blocked replication of the *E. coli* chromosome followed by loss of DNA near the replication origin then terminus. This implies that much of TLD results from an irreversible cell-cycle checkpoint that blocks cell division when single-stranded DNA (the SOS–inducing signal) accumulates and that the rest results from DNA destruction, models for which are presented.

## Introduction

Thymineless death (TLD), the rapid loss of viability in cultures deprived of thymine, occurs in *E. coli*, yeast and human cells (reviewed [1]). Cancer chemotherapeutic drugs methotrexate, 5-fluorouracil (5-FU), and fluorodeoxyuridine, and the antibiotic trimethoprim, work by inducing TLD by targeting thymidylate synthase and/or interfering with *de novo* synthesis of thymidine monophosphate. Whereas 5-FU kills cells both TLD-dependently and TLD-independently (reviewed [2]), newer drugs are being developed that target thymidylate synthase specifically [3]. Despite its relevance to problems of chemotherapy resistance, and although studied extensively, the mechanism(s) responsible for TLD remain unclear.

Work by Sat et al. suggested that TLD in *E. coli* was a form of cell suicide induced by the MazF toxin gene, an RNase that can induce cell death under various stresses coincident with destruction of mRNAs [4],[5] by a mechanism not fully understood. Though intriguing, this is probably not the full story of TLD. Whereas inhibition of transcription by various drugs relieved TLD [6]–[8], MazF is repressed under active transcription by the presence MazE anti-toxin, and becomes available specifically when transcription is inhibited and MazE is degraded [9], such that inhibiting transcription would have been expected to exacerbate TLD (discussed [8]).

TLD also requires proteins involved in homologous recombination (HR) and repair, such as RecF and RecO which load RecA recombinase onto single-strand (ss)DNA [10],[11], RecQ DNA helicase [12],[13], and RecJ exonuclease [11]. TLD is exacerbated in cells lacking the UvrD helicase [14], which acts in nucleotide excision repair (NER), and mismatch repair, and dismantles RecA filaments on single strand DNA, and so opposes HR [15]. The UvrD anti-TLD role appears not to be *via* its role in NER, because NER-defective *uvrA* cells are not TLD hypersensitive [8]. TLD is also exacerbated in cells lacking RecBCD, the main double–strand exonuclease and catalyst of double-strand-break repair by homologous recombination in *E. coli* [10]. Chromosomal abnormalities/damage are associated with TLD in that cells undergoing TLD exhibit DNA breaks [16] and degradation [17]. Further, abnormal DNA structures detected during TLD are reduced in cells lacking RecF, RecJ, RecQ or RecA [18]. Despite this evidence supporting a mechanism for TLD involving HR proteins, surprisingly, RecA, the central HR protein and activator of the SOS DNA-damage response, was reported not to be required for TLD [10],[19]. In these studies non-null *recA* alleles were used: missense mutations *recA1*, *recA13* and *recA56* encode proteins with diminished strand exchange and SOS induction *in vitro* (measured by LexA cleavage), while retaining the ability to bind ssDNA [20]; and *recA99* is an amber nonsense mutation that results in expression of a 7-amino-acid peptide [21],[22]. A single conflicting report using an undefined *recA* allele [23] drew the opposite conclusion, that RecA was involved in TLD. Moreover, the SOS response, which is controlled by RecA, was also reported not to affect TLD [8]. This might have seemed to contradict a previous report that SulA, a protein made only during SOS, promoted TLD [24]. However, that study tested *sulA* effects only in *lon* (protease-negative) cells, which have abnormally high SulA expression, leaving open the possibility that normally (in *lon^+^* cells) SulA and SOS were not involved.

The evidence that numerous HR proteins promote TLD and the conflicting *recA* literature led us to reinvestigate the roles of RecA, HR proteins, and the SOS response in TLD. We show that RecA, is required for much of TLD, and that its major role is *via* the SOS response. We find that the SOS-controlled inhibitor of cell division, SulA, accounts for most of the requirement for the SOS response in TLD, implicating irreversible checkpoint activation, causing a block to cell division, as a major contributor to TLD. We find that HR proteins previously shown to be required for TLD promote TLD by both SOS-dependent and SOS-independent pathways involving chromosome-segregation failure and apparent chromosome-region-specific DNA destruction.

## Results

### Roles of RecA in TLD

**Figure 1:**
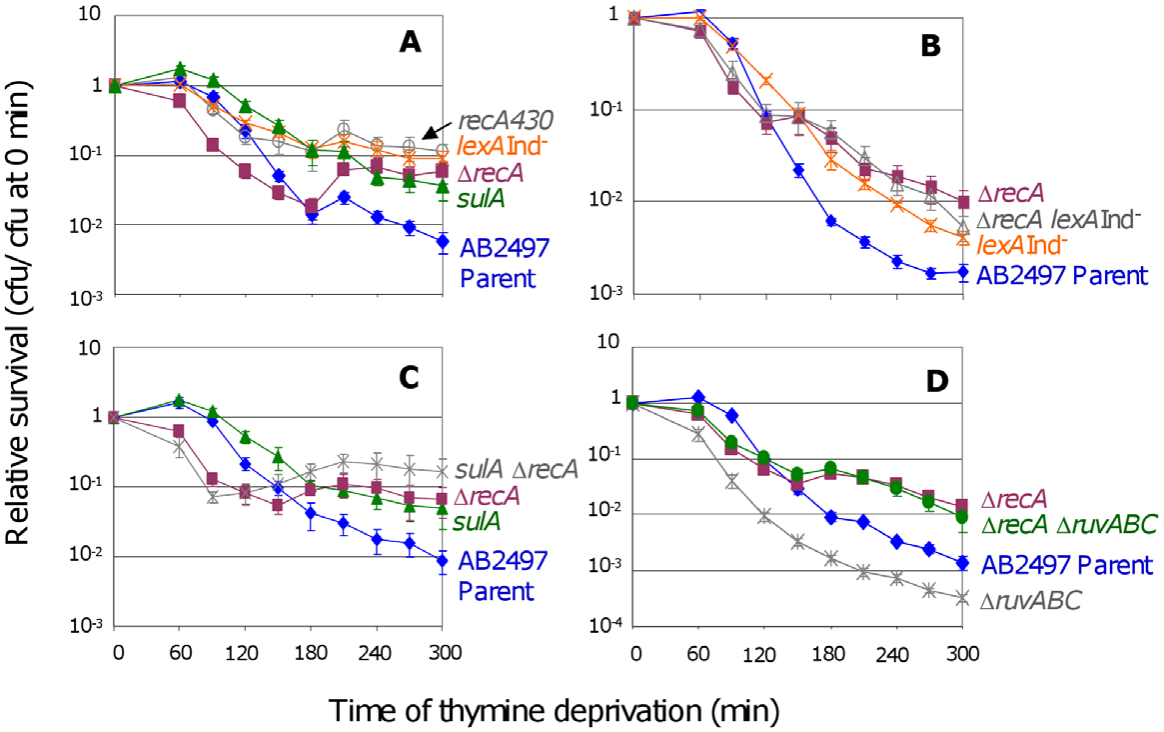
RecA and the SOS response in TLD. (A) Δ*recA* cells (SMR10433, ▪) are significantly more sensitive to thymine deprivation than the isogenic parent (AB2497, ♦) at t≤120 min, but are significantly more resistant at t≥180 min. Inability to induce the SOS response and SulA reduces TLD: *lexA3*(Ind^−^) (SMR10669, 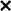) and *recA430* (SMR10668, 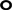) “SOS-off” mutants are not significantly different from Δ*recA* (SMR10433) at t≥240 min, but are significantly more resistant to TLD than their isogenic parent AB2497 at t≥150 min. *sulA* strain (SMR10674, ▴) shows TLD resistance similar to *lexA3*(Ind^−^) and *recA430* “SOS-off” mutants, and significantly greater than the parent at t≥120 min. (B) RecA acts mostly *via* the LexA/SOS pathway of TLD. Δ*recA* (SMR10433, ▪) and Δ*recA lexA3*(Ind^−^) (SMR10912, △) mutants are slightly but not significantly more resistant to TLD than the *lexA3*(Ind^−^) single mutant (SMR10669, 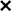), indicating that most of the RecA phenotype is *via* the LexA/SOS pathway. All three mutants are significantly more resistant than their *rec^+^ lex^+^* parent AB2497 (♦). (C) RecA acts mostly in the SulA-dependent TLD pathway. Δ*recA sulA* (SMR10713, 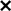) is not significantly different from Δ*recA* (SMR10670, ▪) but shows greater resistance to TLD than *sulA* (SMR10674, ▴) alone. Parental strain AB2497 (♦). (D) RuvABC protect cells from TLD. Δ*ruvABC* (SMR10660, 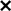) is more sensitive to TLD than its isogenic parent (AB2497, ♦), however Δ*recA* Δ*ruvABC* (SMR11118, •) cells are as resistant to TLD as Δ*recA* (SMR10433, ▪). Mean ± SEM of 5 (A,B) or 3 (C,D) experiments. See Materials and Methods for statistical methods.

In contrast with previous results obtained with non-null *recA* alleles [10],[19], we find that cells carrying a deletion of *recA* are initially more sensitive to thymine deprivation than *rec^+^* cells (Figure 1A, before 180 min.), but are ultimately more resistant to TLD (Figure 1A, after 180 min.). The magnitude of the effect of the *recA* deletion is somewhat variable between experiments (e.g., Figure 1A *versus* Figure 1B), but we observed the same trend in a second genetic background KL742 (Figure S1). Most of the work presented uses the AB2497 genetic background because, first, it has been used commonly in the *E. coli* TLD literature (e.g., [10],[13],[25]), and second, it shows greater sensitivity to thymine deprivation than KL742.

The shape of the Δ*recA* curve (Figure 1A, ▪s) implies that early during thymine deprivation RecA protects against TLD, but at later times RecA contributes to TLD. We do not know why in some instances, Δ*recA* cultures show an increase in colony forming units (cfu) during TLD (e.g., Figure 1C between 120 and 180 min.). Perhaps in the absence of RecA some cells complete an additional round of cell division because some cells lyse, releasing thymine used by the remainder.

In the following section, we show that activation of the SOS DNA-damage response is required for much of TLD. To determine whether the apparent dual roles of RecA in TLD correspond to its two known functions in HR *versus* induction of the SOS response, we examined cells carrying the *recA430* allele, which encodes a RecA protein that is competent for HR but defective for induction of the SOS response [26]. We find that *recA430* cells display the increased TLD resistance seen with the Δ*recA* allele late in thymine starvation, but do not show the increased TLD sensitivity early in TLD seen with the Δ*recA* null allele (Figure 1A). This implies that the early protective role of RecA in TLD is not via SOS-induction, and so could be via HR, whereas the later TLD-promoting role is via SOS induction (discussed below).

MazF is an RNase expressed during stress that leads to programmed cell death (reviewed [27]) and was implicated in TLD [4],[5]. The previously reported requirement for MazF in TLD was variable (complete [4] *versus* 4- to 5-fold [5]) and was not tested in AB2497, the strain used for much previous work on TLD. We wished to understand whether the role of RecA might be, for example, activating expression of MazF. To determine whether the observed role for RecA (Figure 1A, Figure S1) is part of the same pathway as the MazF RNase in TLD, we tested the magnitude of the *mazF* effect in the AB2497 strain used here. We find that Δ*mazF* caused a slight, but insignificant, increase in TLD resistance (Figure S2, see Materials and Methods for statistical methods), indicating that the MazF RNase is not a major mechanism contributing to TLD in this strain. Thus, the role of RecA in promoting TLD is likely to be independent of MazF.

### SOS response and SulA in TLD

RecA functions both in HR and in induction of the SOS response to DNA damage (reviewed [28],[29]). The SOS response is induced when single-stranded (ss)DNA, the SOS-inducing signal, accumulates at sites of DNA damage or blocked replication forks. RecA binds the ssDNA, becomes activated as a co-protease and facilitates auto-proteolytic cleavage of the LexA transcriptional repressor, thus upregulating expression of about 40 damage-inducible SOS genes.

We found that blocking the ability of cells to induce SOS with either of two special “SOS-off” mutations conferred resistance to TLD: *lexA3*(Ind^−^), which encodes an uncleavable LexA/SOS repressor; and *recA430*, the recombination-proficient, SOS-induction-deficient *recA* allele (Figure 1A, orange 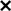
s and grey 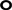
s). We conclude that induction of the SOS response is required for TLD.

Both the *lexA3*(Ind^−^) and *recA430* results reported here contradict a previous report that *lexA3*(Ind^−^) did not affect TLD-sensitivity [8]. Experiments summarized in Figure S3 and legend indicate that the strain used previously contained the *lexA3*(Ind^−^) mutation but additionally carried another genetic element(s) that suppressed the TLD-resistance phenotype.

Addition of an operator-constitutive *recA*o allele, which constitutively produces SOS-induced levels of RecA, to the *lexA3*(Ind^−^) cells did not overcome the resistance to TLD conferred by *lexA3*(Ind^−^) (Figure S4), and is significantly different from the AB2497 parental strain only after 300 minutes of thymine deprivation (p = 0.012). We conclude that SOS-induced levels of a LexA-controlled function other than, or in addition to, RecA is required for TLD.

SulA is an inhibitor of cell division that is expressed only during an SOS response [30]. We find that *sulA* cells are nearly as resistant to TLD as *lexA3*(Ind^−^) or *recA430* cells (Figure 1A), indicating that SulA can account for most or nearly all of the role of the SOS response in TLD. These data imply that a large fraction of TLD results from an irreversible block to cell division caused by SOS induction and SulA expression.

The following data indicate that much of the role of RecA in promoting TLD results from its role in induction of SOS and SulA. First, we see that after 150 min Δ*recA* cells are only slightly more resistant to TLD than *sulA* cells (Figure 1C). Because SulA induction requires RecA [30], this implies that most of the contribution of RecA to TLD is via the same pathway as SulA. Second, *ΔrecA sulA* cells show slightly, but statistically insignificantly greater TLD resistance than the *ΔrecA* single mutant (Figure 1C) indicating, as expected, that *sulA* functions completely in the same pathway as *recA*. Third, *ΔrecA sulA* is slightly but significantly more resistant than the *sulA* single mutant after 210 minutes of thymine deprivation (Figure 1C), indicating that SulA accounts for most, but not all, of the RecA role in TLD. Fourth, Δ*recA* and Δ*recA lexA3*(Ind^−^) cells are also slightly but insignificantly more TLD resistant late in TLD than *lexA3*(Ind^−^) single mutant cells (Figure 1B). This indicates that most of the Δ*recA* phenotype late in TLD occurs via the same pathway (SOS induction) as that blocked in the *lexA3*(Ind^−^) “SOS-off” mutant. The slightly greater TLD resistance of Δ*recA* single mutants and of both *ΔrecA sulA* and Δ*recA lexA3*(Ind^−^) double mutants compared with *sulA* and *lexA3*(Ind^−^) single mutants suggest that there is a small SOS/SulA-independent role for RecA, however, most of the requirement for RecA in TLD appears to occur *via* the same pathway that leads to SOS/SulA induction. This could be because the important role of RecA in TLD is in inducing SOS directly or that, *e.g*., RecA-promoted HR intermediates cause SOS/SulA induction that leads to death, or both.

### Holliday-junction-resolution prevents TLD

As noted above, there is a small RecA-dependent but SOS/SulA-independent component of TLD (previous paragraph, and Figure 1B and 1C). We hypothesized that this segment of TLD might result from “death-by-recombination” (per [31]), caused when interchromosomal HR intermediates (IRIs) accumulate and prevent chromosome segregation, thereby killing cells. Thymine deprivation could lead to ssDNA gaps in replicating DNA. Perhaps while some thymine remains, repair by HR with a sister chromosome is possible and protective, explaining the early part of the Δ*recA* curve; but later in the complete absence of thymine, the cellular capacity to resolve RecA-promoted IRIs might be inhibited and accumulated IRIs could cause chromosome-segregation failure and death (model discussed below).

The RuvABC resolvasome constitutes a major pathway of IRI resolution in *E. coli* [32]. The death-by-recombination hypothesis for TLD predicts that RuvABC would protect cells from TLD by reducing levels of IRIs that cause death. Indeed, we find that deletion of *ruvABC* makes cells more sensitive to TLD (Figure 1D). As predicted, this sensitivity is completely dependent on RecA activity (Figure 1D), implying that accumulation of unresolved RecA-promoted IRIs in cells lacking RuvABC promotes TLD. In support of the interpretation that Δ*ruvABC* exacerbated TLD because of excess unprocessed HJs/IRIs, expression of an unrelated HJ resolvase, RusA, partially compensated for the lack of RuvABC (Figure S5A). RusA is encoded in a cryptic prophage and is expressed if cells carry the *rus-1* mutation, which restores partial resistance to UV light to *ruvC* strains [33]. These experiments do not address whether in wild-type (RuvABC^+^) cells TLD *normally* results from excess IRIs. To test this one would ideally provide *more* resolution capacity than in wild-type cells, and ask whether TLD was reduced. However, because of toxicity effects upon overproduction, interpretations of results from overproduction experiments are inconclusive, and we have not presented those here.

### SOS–dependent and –independent roles of RecF in TLD

Having established a role for RecA in TLD via SulA/SOS response activation, we sought to determine whether other HR proteins previously shown to be required for TLD promote TLD by the same pathway.

**Figure 2:**
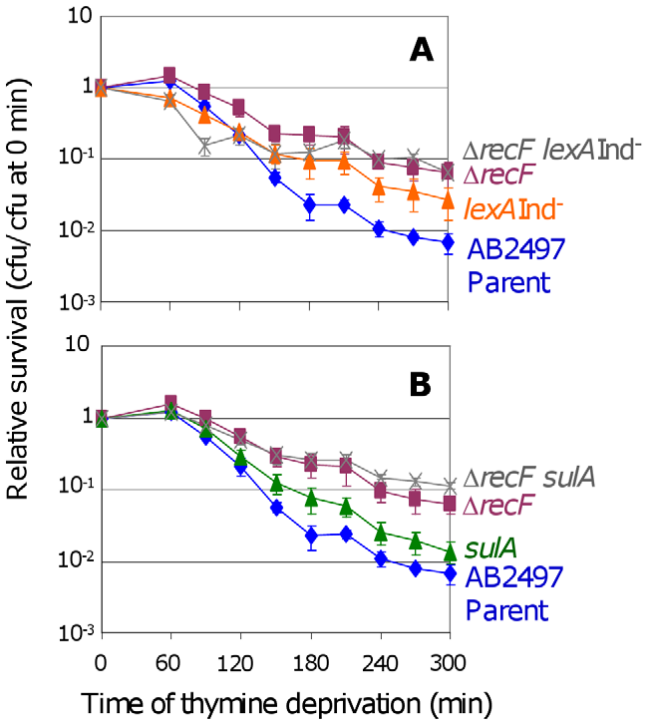
RecF promotes TLD *via* SOS–dependent and SOS–independent pathways. (A) The Δ*recF lexA3*(Ind^−^) (SMR10692) double mutant (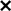) is more TLD resistant than its parent AB2497 (♦) and than *lexA3*(Ind^−^) (SMR10669, ▴), significant at t≥240 min, but not more than the Δ*recF* (SMR10691, ▪) single mutant. (B) Similar results as (A) are seen for the Δ*recF sulA* (SMR10694, 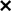) double mutant: greater resistance than AB2497 (♦) and *sulA* (SMR10674, ▴) significant at t≥150 min, and similar resistance to Δ*recF* (SMR10693, ▪). Mean ± SEM of 3 experiments (A,B).

RecF loads RecA onto ssDNA, a precursor to both HR and SOS induction [30], and is required for replication restart [34] apparently *via* activating SOS, in that SOS-constitutive-mutant cells no longer require RecF [35]. We find that both Δ*recF lexA3*(Ind^−^) (Figure 2A) and Δ*recF sulA* (Figure 2B) cells show somewhat greater TLD resistance than *lexA3*(Ind^−^) and *sulA* single mutants, respectively. The difference is significant in both cases (Figure 2 legend). The data imply that most of role of RecF in TLD is in the SOS/SulA-dependent pathway leading to TLD, but that RecF also promotes TLD SOS/SulA-independently either *via* HR or another route.

### RecQ and RecJ promote TLD SOS/SulA- and RecA- independently

**Figure 3:**
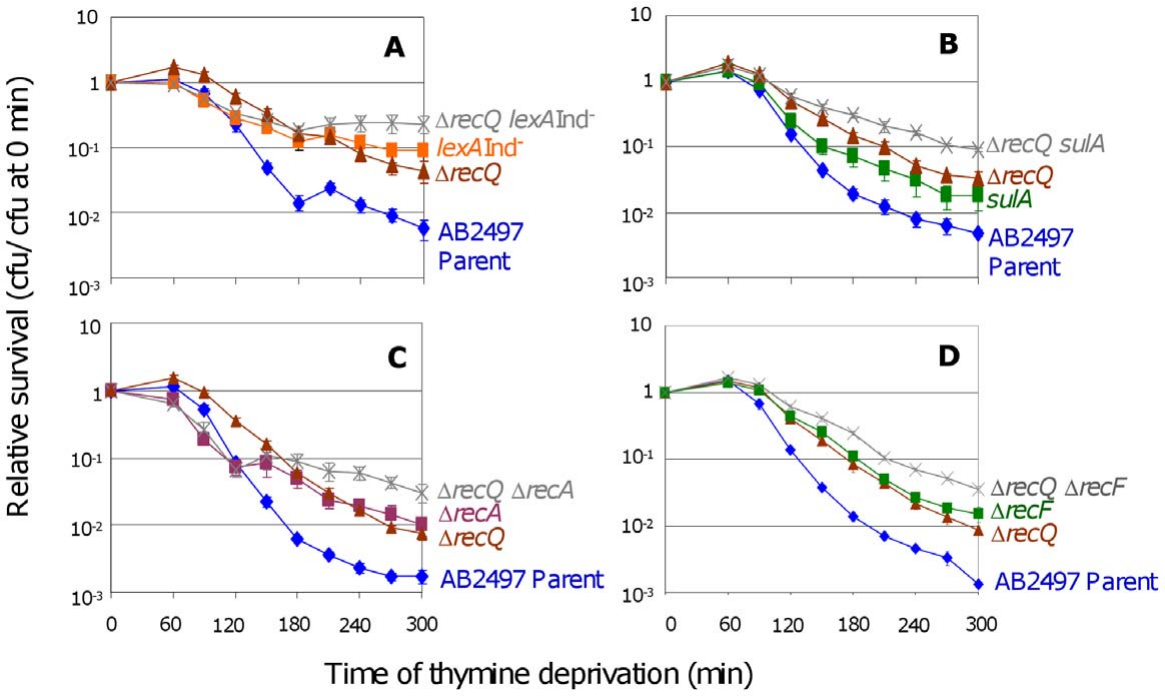
RecQ promotes TLD SOS- and RecA-independently. (A) Additive effects of Δ*recQ* and *lexA3*(Ind^−^) mutations. Δ*recQ lexA3*(Ind^−^) (SMR10683, 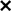) cells show significantly greater TLD resistance than either Δ*recQ* (SMR10436, ▴) at t≥120 min or *lexA3*(Ind^−^) (SMR10669, ▪) at t≥270 min. Parental strain AB2497 (♦). (B) Additive effects of Δ*recQ* and *sulA* mutations. Δ*recQ sulA* (SMR10677, 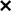) cells show significantly greater TLD resistance than Δ*recQ* (SMR10436, ▴) at t≥180 min and *sulA* (SMR10674, ▪) at t≥120 min. (C) Additive effects of Δ*recQ* and Δ*recA* mutations. Δ*recQ* Δ*recA* (SMR10913, 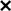) shows significantly greater TLD resistance than Δ*recQ* (SMR10681, ▴) at t≥240 min and Δ*recA* (SMR10433, ▪) at t≥210 min, indicating that RecQ and RecA promote TLD through different pathways. (D) Additive effects of Δ*recQ* and Δ*recF* mutations. Δ*recQ* Δ*recF* (SMR11205, 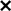) is more resistant to TLD than either Δ*recF* (SMR10691, ▪) or Δ*recQ* (SMR10681, ▴) alone indicating that RecQ and RecF promote TLD through different pathways. Mean ± SEM of 5 (A,C) or 3 (B,D) experiments.

Under some conditions RecQ is required for SOS induction [36]. To test whether the role of RecQ in TLD is *via* SOS/SulA induction, we examined Δ*recQ lexA3*(Ind^−^) (Figure 3A) and Δ*recQ sulA* (Figure 3B) cells. Both double mutants were significantly more resistant than their respective single-mutant controls indicating a wholly or partly additive TLD resistance when both SOS/SulA and RecQ are inactivated. We conclude that RecQ promotes TLD *via* a pathway wholly or partly independent of and additive with the SOS/SulA TLD pathway.

If the sole role of RecQ in TLD were to assist RecA-mediated accumulation of IRIs leading to death by recombination, then loss of RecQ would be expected to provide no further resistance to TLD above that already seen in Δ*recA* cells. However, we observed greater TLD resistance of Δ*recQ* Δ*recA* double mutants than Δ*recA* cells (Figure 3C). Similarly, Δ*recQ* Δ*recF* double mutants showed greater resistance to TLD than Δ*recF* or Δ*recQ* (Figure 3D). We conclude that although RecQ might catalyze death-by-recombination in TLD in a minor pathway, it must also promote TLD by a RecA- RecF-independent, and thus HR-independent mechanism.

**Figure 4:**
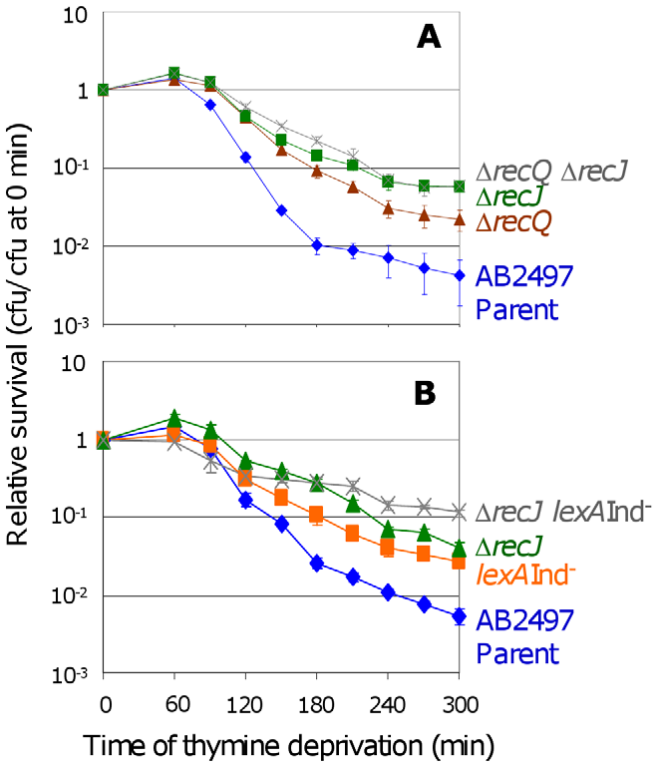
RecJ works with RecQ to promote TLD SOS-independently. (A) RecJ functions in the same TLD pathway as RecQ. Δ*recQ* Δ*recJ* (SMR11198, 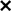) is as resistant to TLD as Δ*recJ* (SMR10695, ▪), but more resistant than Δ*recQ* (SMR10681, ▴). Parental strain AB2497 (♦). (B) Additive effects of *recJ* and *lexA3*(Ind^−^) mutations. *recJ lexA3* (SMR10696; 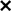) cells show significantly greater TLD resistance than *lexA3* (SMR10669, ▪) at t≥150 min and *recJ* (SMR10695, ▴) at t≥240 min. The wholly or partly additive effects of SOS/SulA with *recQ* and *recJ* mutations indicate that at least part of how RecQ and RecJ promote TLD is independent of the SOS/SulA death pathway. Mean ± SEM of 3 experiments (A,B).

RecJ exonuclease is thought to work closely with RecQ to unwind and degrade nascent DNA at stalled replication forks [36],[37], and *recJ* and *recQ* have similar phenotypes in TLD (Figure 4A and [11]), and also in a “death-by-recombination” pathway in which cells that accumulate unresolved interchromosomal recombination intermediates (IRIs) die from chromosome-segregation failure [31]. We find that the double *recQ recJ* mutant is as resistant to TLD as *recJ* alone (Figure 4A) indicating that these two proteins promote TLD *via* the same pathway. Interestingly *recJ* has a greater resistance to TLD than *recQ* (Figure 4A), possibly because RecQ helicase can create the 5′-ssDNA-end substrate degraded by RecJ exonuclease (e.g., [36],[37]), but RecJ can also degrade 5′-ssDNA ends that arise via means other than RecQ. Like RecQ, RecJ promotes TLD *via* a pathway that is wholly or partly additive with, and thus wholly or partly independent of, SOS induction (Figure 4B); we find that Δ*recJ lexA3*(Ind^−^) cells are significantly more TLD resistant than either Δ*recJ* or *lexA3*(Ind^−^) single mutants. These data show that at least two pathways contribute to TLD, a RecA-, RecF-, and LexA-dependent one requiring SOS/SulA induction and another involving HR proteins RecQ and RecJ without SOS induction or RecA.

### Topoisomerase III plays no role in TLD

Homologues of RecQ have been shown to work with Topoisomerase III in a “dissolvasome” complex to resolve converging replication [38] or recombination intermediates [39]. We tested the possibility that Topoisomerase III was necessary for TLD, similarly to RecQ, but did not find significant resistance to TLD in cells lacking *topB*, the gene encoding Topoisomerase III (Figure S6).

### Chromosome-segregation and -replication defects and DNA loss during TLD

**Figure 5:**
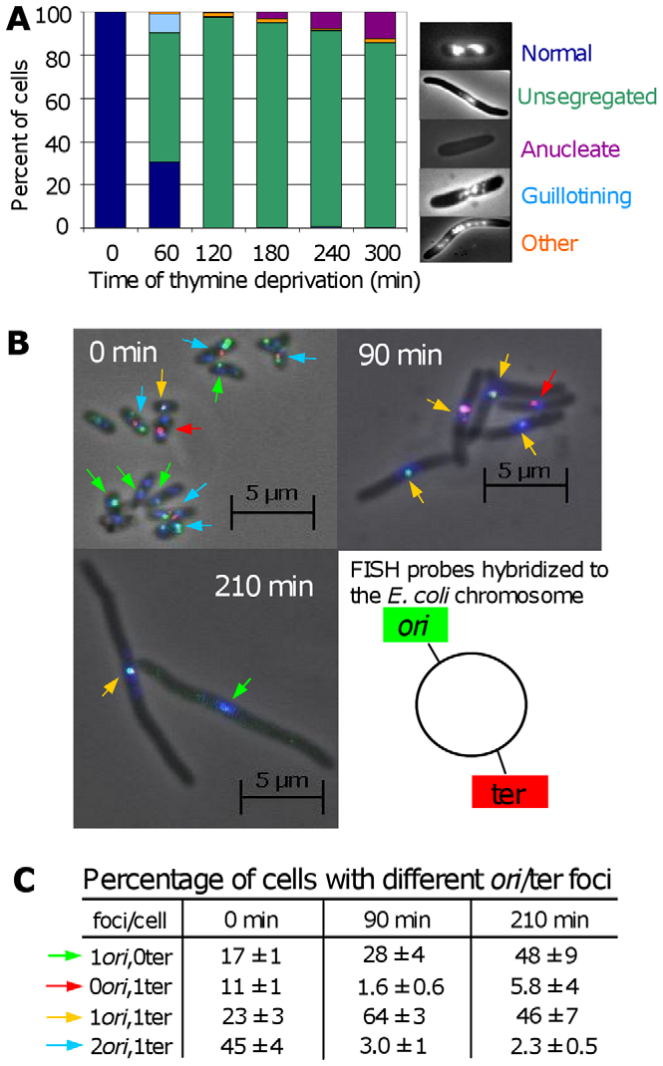
Chromosome segregation and DNA loss during TLD. (A) Chromosome-segregation defects in *E. coli* AB2497 during thymine deprivation. DAPI stained DNA appears as bright masses or nucleoids. ∼300 cells scored per timepoint. (B) Representative FISH of AB2497 cells during TLD. Origins, green foci (and arrows); termini, red foci (and arrows); DNA, blue (DAPI). (C) Percentage of FISH-labeled cells with different numbers *ori* and ter foci showing loss of *ori*-proximal foci early, and ter-proximal foci late during TLD. 1000, 800, and 530 cells scored at 0, 90, and 120 min, respectively. Images in (B) are merges of 3 separate images taken with filters specific for green foci, red foci, and blue DAPI stain; however scoring of types in (B,C) was performed on individual non-merged filtered images in which only green or only red foci were visible. Mean ± SEM of 3 experiments.

We found that the majority of cells undergoing TLD exhibit severe chromosome-segregation defects (Figure 5A). Whereas most cells grown in the presence of thymine appear small and have discreet, segregated nucleoids (bacterial chromosomes), one hour after thymine deprivation most cells appear elongated with a single, small central DNA mass which appears to contain less DNA than normal nucleoids (Figure 5A and 5B 90 min). “Guillotining” of DNA during cell division (see Figure 5A) occurs early during TLD, whereas anucleate cells, which may result from degradation of broken/guillotined DNA or septum formation at the ends of elongated cells, appear later (Figure 5A).

DNA content of the cells undergoing TLD appeared diminished with respect to both normal cells (Figure 5A) and cells dying the death-by-recombination observed previously [31]. We examined chromosome replication and integrity using fluorescent in-situ hybridization (FISH) with probes homologous to the chromosomal replication origin (*ori*) (green) and terminus (ter) (red, Figure 5B). At time 0, cells were small with an average of 2.2±0.1:1 labeled *ori*:ter foci. Per Figure 5C, 45% had 2 *ori* and 1 ter focus, expected in replicating DNA, 23% had 1 of each, and 17% and 11% had only one *ori* or ter focus, respectively. The 17% and 11% with only one *ori* or ter focus presumably reflect the imperfect efficiency of the FISH probes to reveal their targets, as reported previously [31],[40], which is a constant for each probe set against which deviations are compared and normalized ([31],[40], Figure 5C).

**Figure 6:**
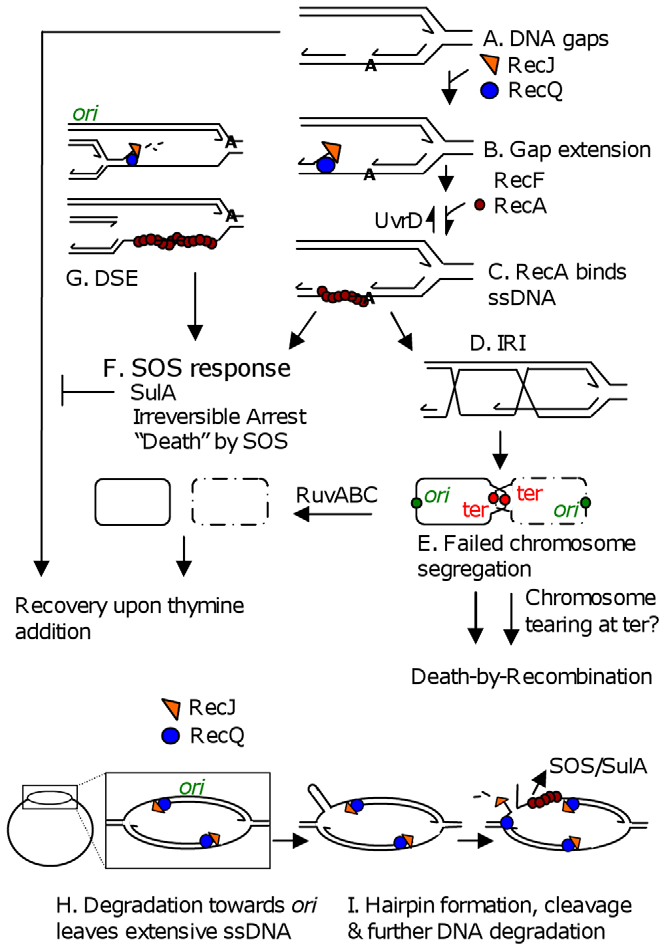
Models for TLD by SOS, death-by-recombination, and RecQ/J-promoted DNA destruction. (A) Gaps in DNA result from insufficient thymine. (B) Gap extension by RecQ and RecJ. (C) RecF-assisted RecA loading at ssDNA gaps promotes strand exchange and IRI formation. (D) Some portion of IRIs can be resolved by RuvABC to yield segregated chromosomes. (E) “Death-by-recombination,” a death caused by failed chromosome segregation, occurs when more IRIs accumulate than can be processed by Ruv and other IRI-resolution pathways [31]. (F) SOS induction and SulA production create an irreversible cell-cycle checkpoint which prevents cells from recovering upon addition of thymine. Note that all ssDNA substrates drawn, including those in *G*, *H* and *I* could lead to SOS-induction. (G) RecQ/J-mediated DNA fragmentation, a possible mechanism for the RecA-independent contribution of RecQ/J to TLD. This degradation towards the *ori* from stopped forks along the chromosome, then (not shown) RecBCD-mediated degradation of the double-stranded DNA end back, past *ori*, to the next stalled fork, would lead to specific loss of *ori*-containing DNA. (H) RecQ/RecJ-promoted DNA destruction. Nascent-strand removal from stopped forks to *ori* and past to the next stopped fork, might be used to restore arrested replication bubbles to the duplex state, but creates extensive ssDNA. (I) Breakage of an old strand in regions of extensive ssDNA, shown here to occur if a hairpin forms and is cleaved by a hairpin endonuclease (but possible with other secondary structures), opens the whole chromosome up to degradation by RecQ and RecJ. This model accounts for apparent loss of first *ori-* then ter-containing DNAs. Lines, strands of DNA except in bottommost schemes (F, and H left) in which solid and dashed circles represent whole bacterial chromosomes; arrow heads, 3′ ends; IRI, interchromosomal recombination intermediate.

The profile of *ori* and ter foci changed dramatically with prolonged thymine deprivation (Figure 5C). At 90 min (Figure 5C), only 3% had 2 *ori*:1 ter, whereas 64% had 1 of each. Although it is formally possible that many chromosomes completed replication but did not re-initiate, this is highly unlikely given the absence of thymine. A more likely explanation is that replication halted mid-chromosome. In this second (more likely) instance, the subsequent shift from the majority of cells containing 2 *ori* and 1 ter to the majority containing a single *ori* and ter over the first 90 min of thymine deprivation may indicate that *ori-*containing DNA was specifically lost or destroyed. Significantly, those with 1 *ori*:0 ter focus increased to 28%, implying loss of *ter*-containing DNA. Supporting this interpretation, the fraction with 1 ter:0 *ori* decreased to 1.6%. This pattern was more pronounced at 210 min (Figure 5C), at which time 2 *ori*:1 ter cells fell further to 2.3%; 1 *ori*:1 ter cells dropped to 46%; while cells with 1*ori*:0ter increased correspondingly to 48%. During normal segregation of daughter chromosomes, first, *ori'*s segregate to the distal cell poles away from the cell-division septum while the ter sequences localize at the septum and are replicated and segregated last [40] (illustrated Figure 6E). During TLD, first, all of the foci stayed mid-cell, where the cell-division septum would form in nonarrested cells (Figure 5B); second, the number of foci per cell is fewer than normal (Figure 5B and 5C); and third, the accumulation of 1 *ori*:1 ter cells replacing 2 *ori*: 1 ter cells, followed by depletion of 1 *ori*:1 ter and concurrent accumulation of 1 *ori*:0 ter cells implies that chromosomes either completed replication then lost their ter sequences, or, more likely given the general DNA reduction seen (Figure 5A and 5B), lost one of their two *ori*s, then subsequently lost ter-containing DNA. We failed to observe a significant fraction of cells containing a single ter and no *ori* (discussed below). The apparent degradation of DNA near ter (which is probably preceded by degradation near one of the two *ori*s) could be caused by chromosome tearing as cells try to segregate unresolved chromosomes, perhaps unsegregated because of IRIs per death-by-recombination models (Figure 6E), or by RecQ/J-promoted DNA degradation, discussed below.

## Discussion

The data presented establish a prominent role for RecA, the SOS response and the SOS-controlled SulA inhibitor of cell division in TLD. They further show that at least three pathways of TLD operate concurrently with a remarkable pattern of chromosome-segregation failure and chromosome-region-specific loss of FISH-detectable foci, in which first apparent replication-origin-containing then terminus-proximal DNA disappeared.

### Death by SOS

First, a major TLD pathway, constituting ≥1 of the 2–3 logs of loss of colony-forming ability observed by 300 min of thymine starvation, is attributable to RecA- and RecF-dependent activation of the SOS DNA-damage response turning on the SulA inhibitor of cell division (Figure 1, Figure 2). This implies, surprisingly, that a significant fraction of TLD results from an irreversible cell-cycle checkpoint such that when returned to medium with thymine in the cfu assay, cell division does not resume. Simply removing *sulA* allowed these cells to form colonies (Figure 1, Figure 2, Figure 3), as if many of the underlying DNA problems that caused SOS induction and SulA expression were not themselves lethal. Irreversible SOS-induction causing apparent cell stasis or senescence has been reported previously in a study from one of our laboratories of spontaneous SOS induction in growing *E. coli* populations which showed that only about 30% of spontaneously SOS-induced cells recover to a proliferating state [41]. The discovery that at least a log of TLD results from inhibition of cell division raises the possibility that some TLD might reflect cell stasis rather than death. A model for the SOS/SulA dependent component of TLD is shown in Figure 6F. SOS is activated when RecA binds ssDNA [30]. In Figure 6 we consider potential sources of ssDNA that might activate SOS during TLD, some of which would not otherwise kill cells (discussed below).

### Death by recombination

A minor second TLD pathway appeared to require RecA but not SulA (Figure 1C) and so might reflect a lethal role of HR. In Figure 6A–6E we consider a “death-by-recombination” model for this component of TLD, based on the observations of death by recombination in Holliday-junction-resolution-defective cells ([31] and references therein). In it we hypothesize that ssDNA gaps caused by inability to replicate in the absence of thymine provoke the RecQ, J, F, A-dependent initiation of HR with a sister chromosome (Figure 6A–6E) creating interchromosomal recombination intermediates (IRIs, Figure 6D and 6E). IRIs are normally resolved by RuvABC allowing chromosome separation (Figure 6D) [32], but we suggest that when the number of gaps and resulting IRIs exceeds resolution capacity, their failure to be resolved will cause death by failed chromosome segregation (Figure 6E). Death by failed chromosome segregation caused by excessive IRI accumulation (“death-by-recombination”) was seen in cells lacking Ruv resolution and UvrD anti-RecA proteins [31] and cells lacking UvrD and RecG Holliday-junction-processing proteins [42], and, like TLD, required RecA, RecF, RecQ and RecJ (SOS independently). As predicted by this model, TLD is associated with failed chromosome segregation (Figure 5) and is exacerbated by removal of RuvABC (Figure 1D), implying that a mechanism like this can occur at least in Ruv-deficient cells. A possible death-by-recombination component of TLD might underlie the minor RecA-dependent SulA-independent fraction of TLD (Figure 1C).

### Death by RecQ and RecJ

Yet a third TLD pathway requires RecQ and RecJ but is dependent upon neither RecA nor SOS induction, and thus is also HR-independent (Figure 3, Figure 4).

In Figure 6G–6I we suggest two HR-independent ways by which RecQ and RecJ could cause TLD and the DNA destruction suggested by our cytological and FISH results (Figure 5). In Figure 6G, RecQ helicase and RecJ 5′ exonuclease are shown degrading DNA at a 5′ end at a replication fork lagging strand [36], leading to DNA fragmentation when the next fork upstream is reached. Because this mechanism degrades newly replicated DNA from a stopped fork towards the *ori*, this might cause the observed loss of *ori*-containing foci early during TLD (Figure 5B and 5C, assuming degradation of the double-strand DNA end created, see Figure 6G), and could explain RuvC-independent linearization of *E. coli* chromosomes during TLD reported by Guzman and colleagues [43], but does not explain ter-specific DNA loss.

Similarly, when replication forks stop in thymine-starved cells, RecQ 5′ helicase and RecJ 5′ single-strand-dependent exonuclease might degrade DNA extensively from the forks' 5′-ending lagging strands back towards the *ori* (Figure 6H), removing both nascent strands from arrested replication bubbles so that cells unable to complete replication return their chromosomes to a simple double-stranded circular starting point allowing re-initiation of replication later, when replication precursors are available (not an apparently death-promoting activity).

Although this appears to predict only *ori*-proximal DNA loss, extensive nascent-strand degradation would expose long tracts of ssDNA, which would induce SOS and might also be susceptible to further breakage upon exposure of secondary-structure-forming sequences in the extensive ssDNA regions (Figure 6I). Digestion of secondary structures would break an “old” strand in these replication bubbles which would then open up the whole chromosome to degradative activities, including the ter (Figure 6I). This might underlie the initial loss of *ori*-containing FISH foci, and later loss of terminus-proximal FISH foci because after an old strand is broken, single-strand degradation can pass a stopped fork and proceed towards the terminus (Figure 6I, right). Although simple removal of nascent strands (Figure 6H) would be expected not to be lethal, breaking an old strand followed by chromosome degradation (Figure 6I) could be lethal. Both models 6G and 6I can explain why there is first loss of only one of two *ori* foci.

Another possibility for ter-specific DNA loss is that chromosome dimers formed by HR that are not resolved will accumulate as shown in Figure 6E, with ters at the cell-division septum and *ori*'s away from it [40]. Tearing of unresolved chromosomes might be expected to occur terminus proximally and this could set off degradation specific to the ter region. Perhaps chromosome dimers formed by HR, which are usually resolved at the septum by XerCD [44], cannot be resolved when cell division is blocked by SulA, and this could result in such tearing (Figure 6E).

### Other TLD pathway(s)

In addition to the TLD pathways listed, our data indicate that at least one more must operate because *recA recQ* cells which are defective for SOS, HR, and the SOS/HR-independent roles for RecQ in TLD, still suffer ≥1 log of TLD by 300 min (Figure 4C).

### Cancer chemotherapies and resistance

Thymineless death is the mode of action of important cancer chemotherapeutic drugs methotrexate, 5-fluorouracil and 5-fluorodeoxyuridine, as well as the antibiotic trimethoprim. The results presented here catalogue a series of proteins and pathways that if disrupted could be expected to confer some level of resistance to those drugs in bacteria and humans. Humans have several RecA homologues including RAD51, whose function in double-strand-break repair by HR is disrupted in *BRCA*-defective cells including in some breast and ovarian cancers (reviewed [45]). Humans possess five RecQ homologues, defects in three of which are known to be associated with cancer-predisposition syndromes (reviewed [46]), any of which might also be defective in sporadic cancers. Cancers with the homologues and analogues of these bacterial DNA repair pathways disrupted might be resistant to TLD, and so to treatment with TLD-inducing drugs. Similarly disruption of the eukaryotic DNA-damage responses and checkpoints might also confer resistance as seen for the SOS response here. The DNA repair, replication and metabolism pathways are very well conserved from bacteria to humans (reviewed [30]) making application of these mechanisms to human cancer treatment plans and investigations practical and imperative.

## Materials and Methods

### Strains and TLD assays

Origins of strains used in this study are given in Table S1. P1 transductions were as described [47]. TLD experiments were as described [4] with minor variations. Thymine auxotrophs were grown at 37°C with shaking in M9 minimal medium with 50 µg/ml thymine, 0.1% glucose and 0.5% casamino acids, and for strains containing pGB2 or pGBruvABC, 100 µg/ml spectinomycin. Saturated cultures were diluted 25-fold into the same medium and grown to early/mid-log (OD_450_ of 0.5). 1.0 ml samples were centrifuged, washed twice with M9 saline solution, and resuspended in 2.0 ml of M9 with glucose and casamino acids (no thymine), then returned to 37°C, with shaking, for up to five hours with aliquots taken at intervals for cfu assays on LBH thymine plates. Cfu were scored on a Microbiology International ProtoCOL colony counter after 24 h at 37°C. Longer incubations verified that all cfu were apparent at 24 h. Because the absolute extent of killing varied widely between experiments, whereas the relative effect of the mutations used did not, data presented show curves that are means of sets of independent experiments in which absolute extents of killing were similar.

### Statistical analyses

Error bars represent ± one SEM of ≥3 independent experiments. Statistical analyses were performed using SigmaStat. For TLD assays significance was determined as p<0.05 using repeated measure ANOVA to analyze the curve data, and Tukey post-hoc analysis.

### Microscopy

Chromosome-segregation analyses were as described [31] with minor changes. Cells were fixed by adding an equal volume of PBS with 4% paraformaldehyde for 10 min at room temperature and 20 min on ice, washed three times with cold PBS and stored in an appropriate volume of PBS. Cells were stained with 4′,6-diamidino-2-phenylindole (DAPI; 1 µg/ml), placed on slides, and photographed with an Olympus B×51 microscope equipped with an Uplan Fluorite 100× oil objective, DAPI filter (U-N31000, Olympus), and an Olympus MagnaFire CCD digital camera.

### Fluorescence in situ hybridization

FISH was as described [31]. Probes were 6 kb DNA fragments PCR amplified (Phusion DNA polymerase, New England Biolabs) from MG1655 DNA. Primers for the *ori* and ter probes were as described [40]. Probes were visualized using a Zeiss Axio Imager microscope equipped with 100× oil immersion objective, DAPI filter, Oregon Green filter, Rhodamine filter, and Hamamatsu EMCCD camera. Foci were scored on each channel prior to RGB merging of the images. Images were processed using Axiovision digital image processing software and ImageJ.

## Supporting Information

Figure S1 RecA is required for TLD in the KL742 strain background. Δ*recA* cells (SMR10432, ▪) are significantly more resistant to TLD than KL742 (♦) at t≥180 min, and Δ*recQ* cells (SMR10435, ▴) are significantly more resistant at t≥90 min. The results recapitulate those shown in Figure 1A, Figure 3A using the AB2497 strain background. Mean ± SEM of 3 experiments.
(0.09 MB TIF)

Figure S2 MazF is not the predominant cause of TLD in the AB2497 strain background. A strain lacking the MazF toxin (SMR10685, ▪) of the MazEF toxin/antitoxin pair is slightly, but not significantly, more resistant to TLD than the parental strain (♦). Mean ± SEM of 3 experiments.
(0.09 MB TIF)

Figure S3 The *lexA3*(Ind^−^) mutation causes TLD resistance in the HL353 strain background. This was observed when the strain HL353 *lexA3*(Ind^−^) was reconstructed (SMR10675, ▴), but not with the originally published construction: HL354 (▪). SMR10675 is significantly different from HL353 (♦) at t≥180 min. Mean ± SEM of 3 experiments. To understand why Morganroth and Hanawalt saw no TLD-resistance in a *lexA3*(Ind^−^) strain relative to its *lexA^+^* parent [8], whereas we observed TLD resistance of both a *lexA3*(Ind^−^) strain and *recA430* strain relative to their isogenic *lexA^+^ recA^+^* parent (Figure 1A), we first repeated their result with their strains HL353 (Parental) and HL354 (*lexA*Ind^−^) (this figure). Next, we reintroduced the *lexA3*(Ind^−^) allele by phage P1-mediated transduction into the HL353 genetic background used by Morganroth and Hanawalt, thus creating strain SMR10675. We observed that SMR10675, but not the originally published *lexA3*(Ind^−^) strain HL354, was TLD resistant (this figure), confirming our finding that an inducible SOS response is required for TLD. We sequenced the *lexA* gene and verified the presence of the *lexA3*(Ind^−^) mutation (G to A at position 355 [Markham, et al]) and the absence of any other mutation in the *lexA* gene or ≥500 bp up- or downstream of *lexA* in all three putative *lexA3*(Ind^−^) strains: ours in the AB2497 strain background (SMR10669), SMR10675 and HL354. Because the *lexA3*(Ind^−^) allele confers TLD-resistance in both genetic backgrounds, including when moved afresh into HL353, because a different SOS-off mutation, *recA430*, also confers TLD resistance, and because SulA is required for TLD (Figure 1A and 1C) and is expressed only during SOS [Courcelle, et al], we conclude that induction of SOS is required for TLD. It seems most likely that some other, unknown mutation(s) is present in HL354 which suppresses the TLD-resistance phenotype conferred by *lexA3*(Ind^−^) in that strain. [Markham BE, Little JW, Mount DW (1981) Nucleotide sequence of the lexA gene of *Escherichia coli* K-12. Nucleic Acids Res 9: 4149-4161.] [Courcelle J, Khodursky A, Peter B, Brown PO, Hanawalt PC (2001) Comparative gene expression profiles following UV exposure in wild-type and SOS-deficient *Escherichia coli*. Genetics 158: 41-64.]
(0.10 MB TIF)

Figure S4SOS–induced levels of RecA do not compensate for an uninducible SOS/LexA regulon in TLD. The *recAo* strain SMR10673 (▴) was not significantly different from the isogenic parent AB2497 (♦) except for at 300 minutes of thymine deprivation (p = 0.012) and *recAo lexA3*(Ind^−^) cells (SMR10676, 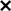) were no more TLD sensitive than *lexA3*(Ind^−^) (SMR10669, ▪) or *sulA* (SMR10674, •) cells. Mean ± SEM of 3 experiments.(0.13 MB TIF)

Figure S5RusA expression partially reverses the hyper-TLD-sensitivity of Δ*ruvABC* cells. (A) The RusA resolvase, expressed in *rus-1* cells, partially restores TLD resistance to Δ*ruvABC* cells. In the Δ*ruvABC* (SMR10689, ▴) background, the *rus-1* allele (SMR10690, 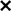) increased resistance to TLD, but in the *thy* ^−^ *rus* ^+^ parental background (SMR10687, ▪) activating RusA *via rus-1* mutation (SMR10686, ♦) did not have a significant effect. We cannot rule out the possibility that the lack of effect in Ruv^+^ cells is due to an inability of RusA to function when RuvABC are present (*in vitro*, RusA was inhibited by RuvA [McGlynn, et al]). Also, there is no reason to believe that *rus-1* creates more resolution capacity than in wild-type cells, such that restoration to Ruv^+^ levels might be expected. (B) Possible RusA effects on TLD are not masked by SulA. Similar results to those in (A) are obtained even when RusA is activated in the absence of SulA. RusA activation partially suppressed the TLD hypersensitivity of Δ*ruvABC sulA* cells (SMR10719, •, and SMR10718, ▴, respectively), but activating RusA in the absence of SulA (SMR10717, ▪) conferred no additional TLD-resistance over that conferred by *sulA* alone (SMR10716, ♦). This rules out the possibility that SulA expression might mask increased TLD-resistance of *rus-1* cells by preventing cell division. Means ± SEM of 3 experiments (A,B). [McGlynn P, Lloyd RG, Marians KJ (2001) Formation of Holliday junctions by regression of nascent DNA in intermediates containing stalled replication forks: RecG stimulates regression even when the DNA is negatively supercoiled. Proc Natl Acad Sci U S A 98: 8235-8240.](0.20 MB TIF)

Figure S6 Topoisomerase III is not required for TLD. Cells lacking *topB* (SMR10672, ▪) are not significantly more resistant to TLD than their isogenic parental strain (AB2497; ♦), indicating that Topoisomerase III is not required for the RecQ-pathway of TLD in *E. coli*. Mean ± SEM of 3 experiments.
(0.09 MB TIF)

Table S1 *E. coli* strains and plasmids used.
(0.26 MB DOC)
