# Supplementary figures and images for: Role of RecA and the SOS Response in Thymineless Death in Escherichia coli
Source: PLoS Genet. 2010 Mar 5;6(3):e1000865. doi: 10.1371/journal.pgen.1000865 (PMC2832678; doi:10.1371/journal.pgen.1000865)

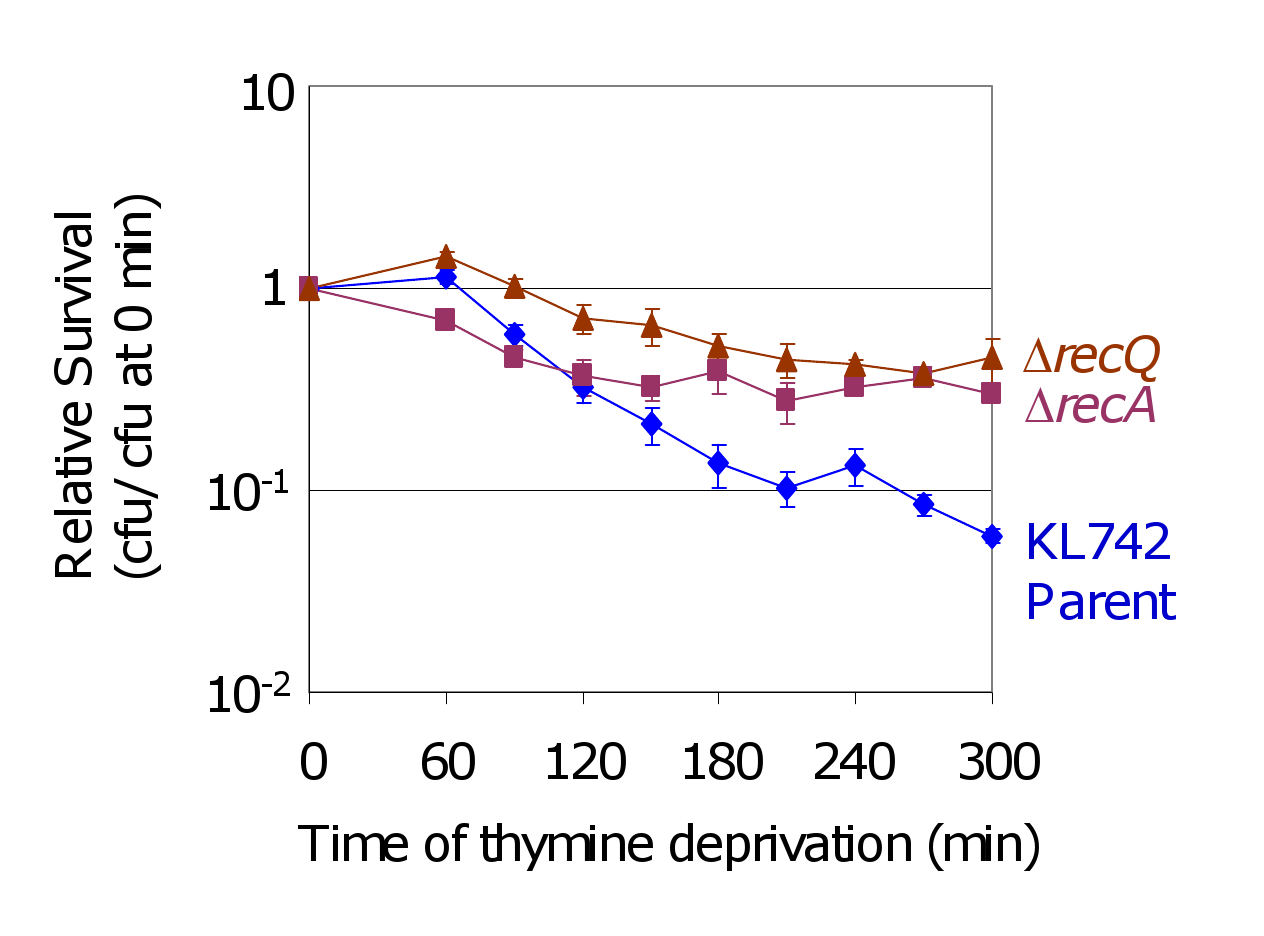

Supplement: Figure S1 — RecA is required for TLD in the KL742 strain background. ΔrecA cells (SMR10432, ▪) are significantly more resistant to TLD than KL742 (♦) at t≥180 min, and ΔrecQ cells (SMR10435, ▴) are significantly more resistant at t≥90 min. The results recapitulate those shown in Figure 1A, Figure 3A using the AB2497 strain background. Mean ± SEM of 3 experiments. (0.09 MB TIF) [file pgen.1000865.s001.tif]

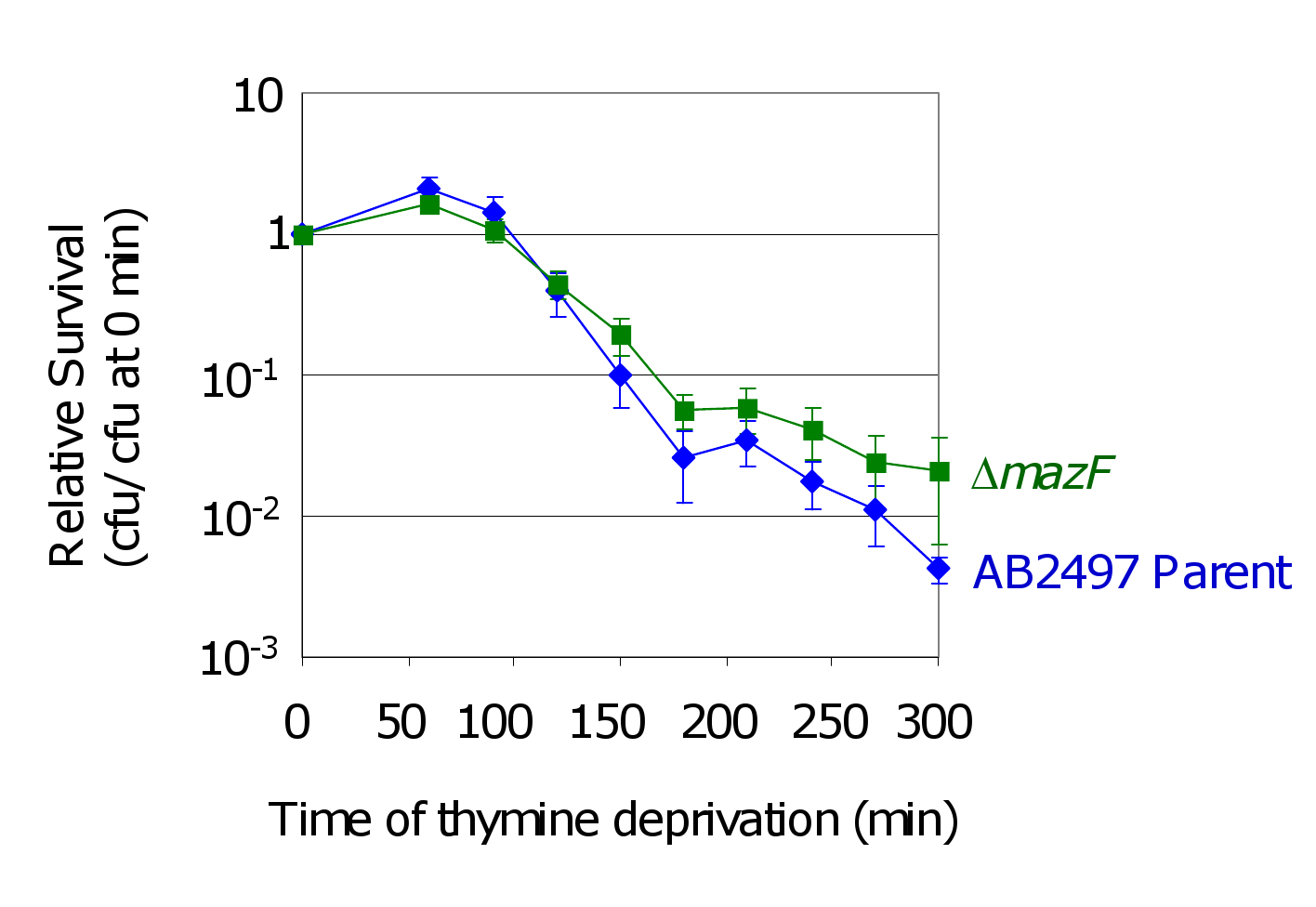

Supplement: Figure S2 — MazF is not the predominant cause of TLD in the AB2497 strain background. A strain lacking the MazF toxin (SMR10685, ▪) of the MazEF toxin/antitoxin pair is slightly, but not significantly, more resistant to TLD than the parental strain (♦). Mean ± SEM of 3 experiments. (0.09 MB TIF) [file pgen.1000865.s002.tif]

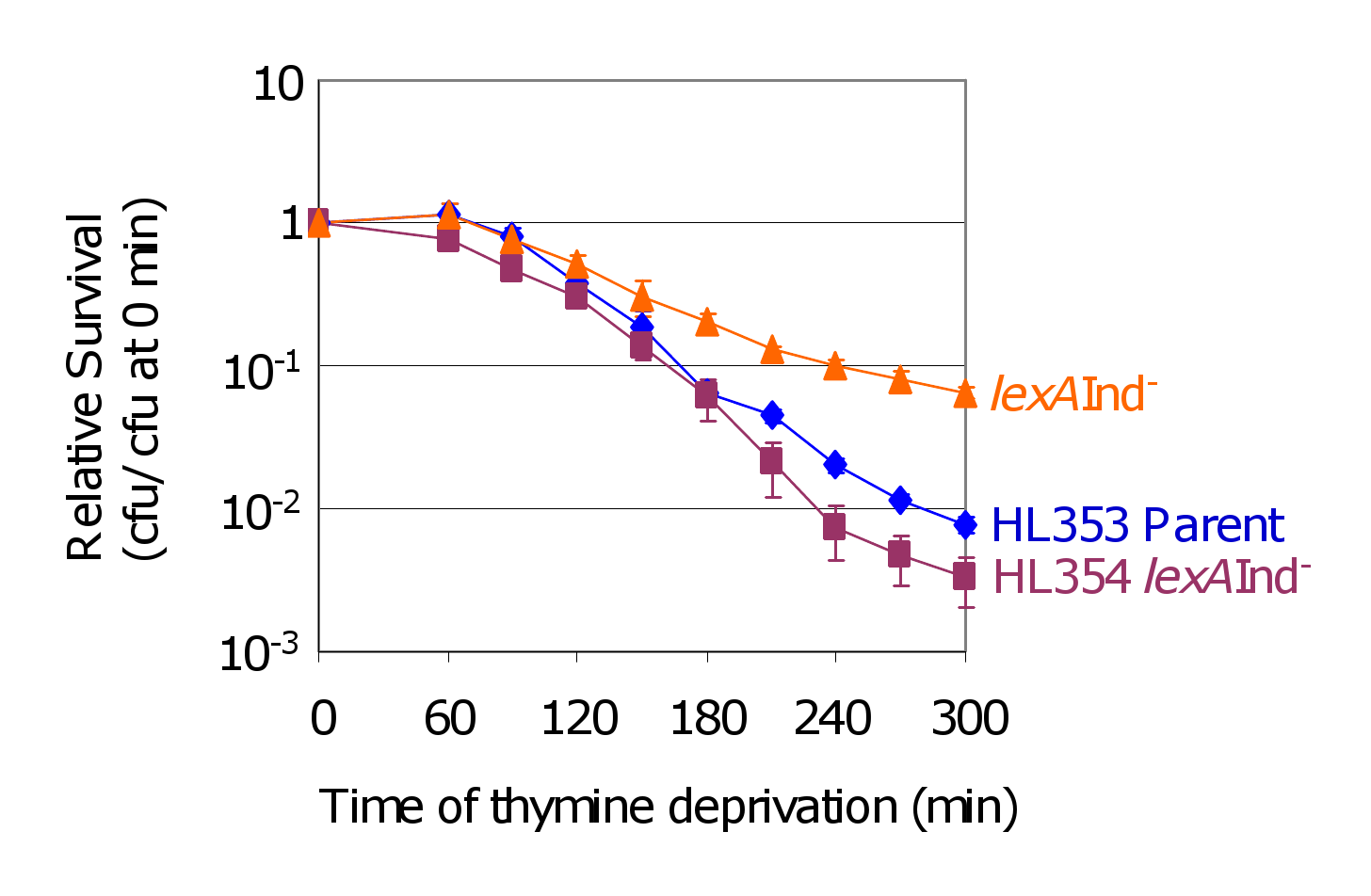

Supplement: Figure S3 — The lexA3(Ind−) mutation causes TLD resistance in the HL353 strain background. This was observed when the strain HL353 lexA3(Ind−) was reconstructed (SMR10675, ▴), but not with the originally published construction: HL354 (▪). SMR10675 is significantly different from HL353 (♦) at t≥180 min. Mean ± SEM of 3 experiments. To understand why Morganroth and Hanawalt saw no TLD-resistance in a lexA3(Ind−) strain relative to its lexA+ parent [8], whereas we observed TLD resistance of both a lexA3(Ind−) strain and recA430 strain relative to their isogenic lexA+ recA+ parent (Figure 1A), we first repeated their result with their strains HL353 (Parental) and HL354 (lexAInd−) (this figure). Next, we reintroduced the lexA3(Ind−) allele by phage P1-mediated transduction into the HL353 genetic background used by Morganroth and Hanawalt, thus creating strain SMR10675. We observed that SMR10675, but not the originally published lexA3(Ind−) strain HL354, was TLD resistant (this figure), confirming our finding that an inducible SOS response is required for TLD. We sequenced the lexA gene and verified the presence of the lexA3(Ind−) mutation (G to A at position 355 [Markham, et al]) and the absence of any other mutation in the lexA gene or ≥500 bp up- or downstream of lexA in all three putative lexA3(Ind−) strains: ours in the AB2497 strain background (SMR10669), SMR10675 and HL354. Because the lexA3(Ind−) allele confers TLD-resistance in both genetic backgrounds, including when moved afresh into HL353, because a different SOS-off mutation, recA430, also confers TLD resistance, and because SulA is required for TLD (Figure 1A and 1C) and is expressed only during SOS [Courcelle, et al], we conclude that induction of SOS is required for TLD. It seems most likely that some other, unknown mutation(s) is present in HL354 which suppresses the TLD-resistance phenotype conferred by lexA3(Ind−) in that strain. [Markham BE, Little JW, Mount DW (1981) Nucleotide sequence of the lexA gene of Escher [file pgen.1000865.s003.tif]

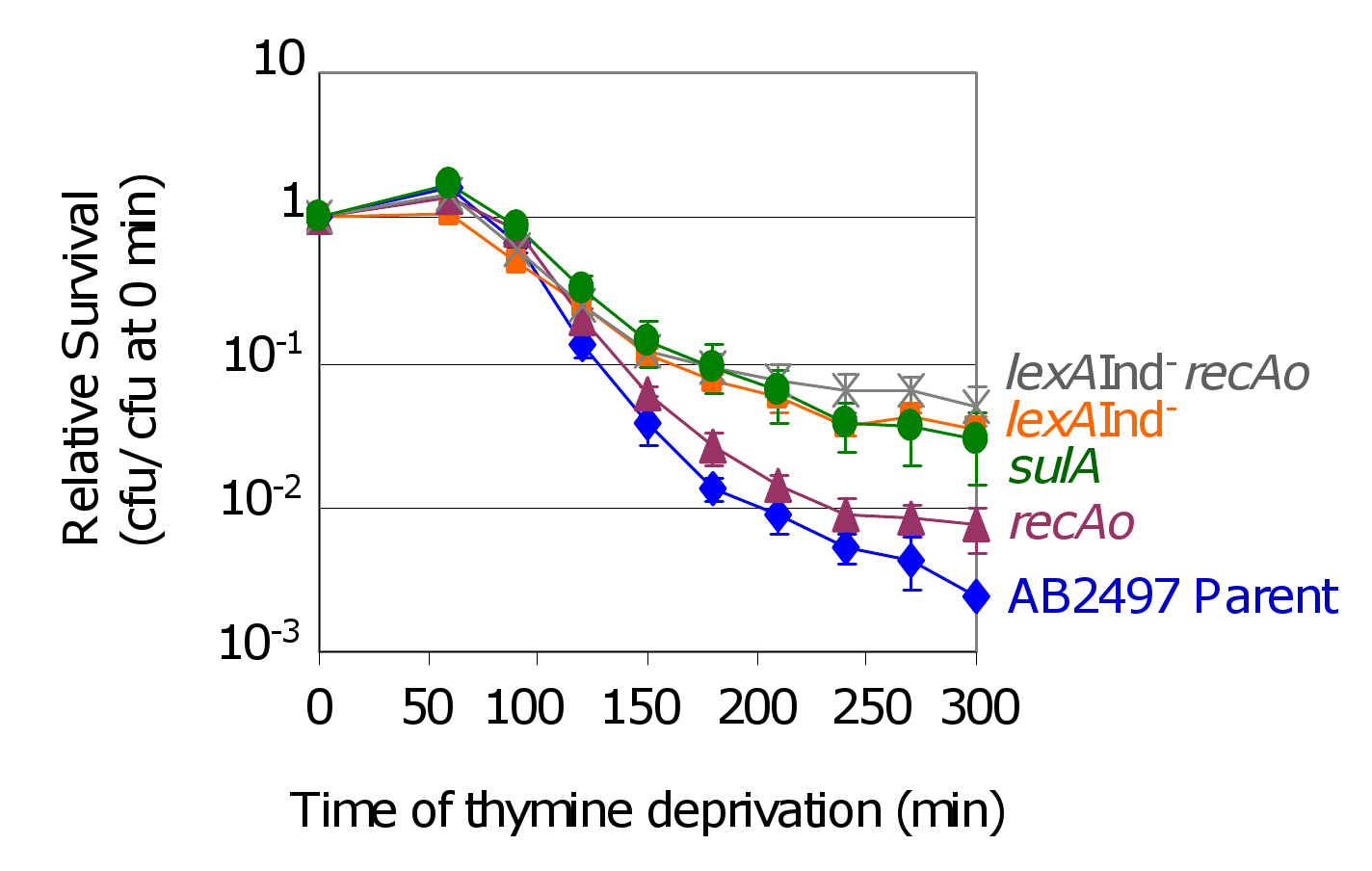

Supplement: Figure S4 — SOS–induced levels of RecA do not compensate for an uninducible SOS/LexA regulon in TLD. The recAo strain SMR10673 (▴) was not significantly different from the isogenic parent AB2497 (♦) except for at 300 minutes of thymine deprivation (p = 0.012) and recAo lexA3(Ind−) cells (SMR10676, ) were no more TLD sensitive than lexA3(Ind−) (SMR10669, ▪) or sulA (SMR10674, •) cells. Mean ± SEM of 3 experiments. (0.13 MB TIF) [file pgen.1000865.s004.tif]

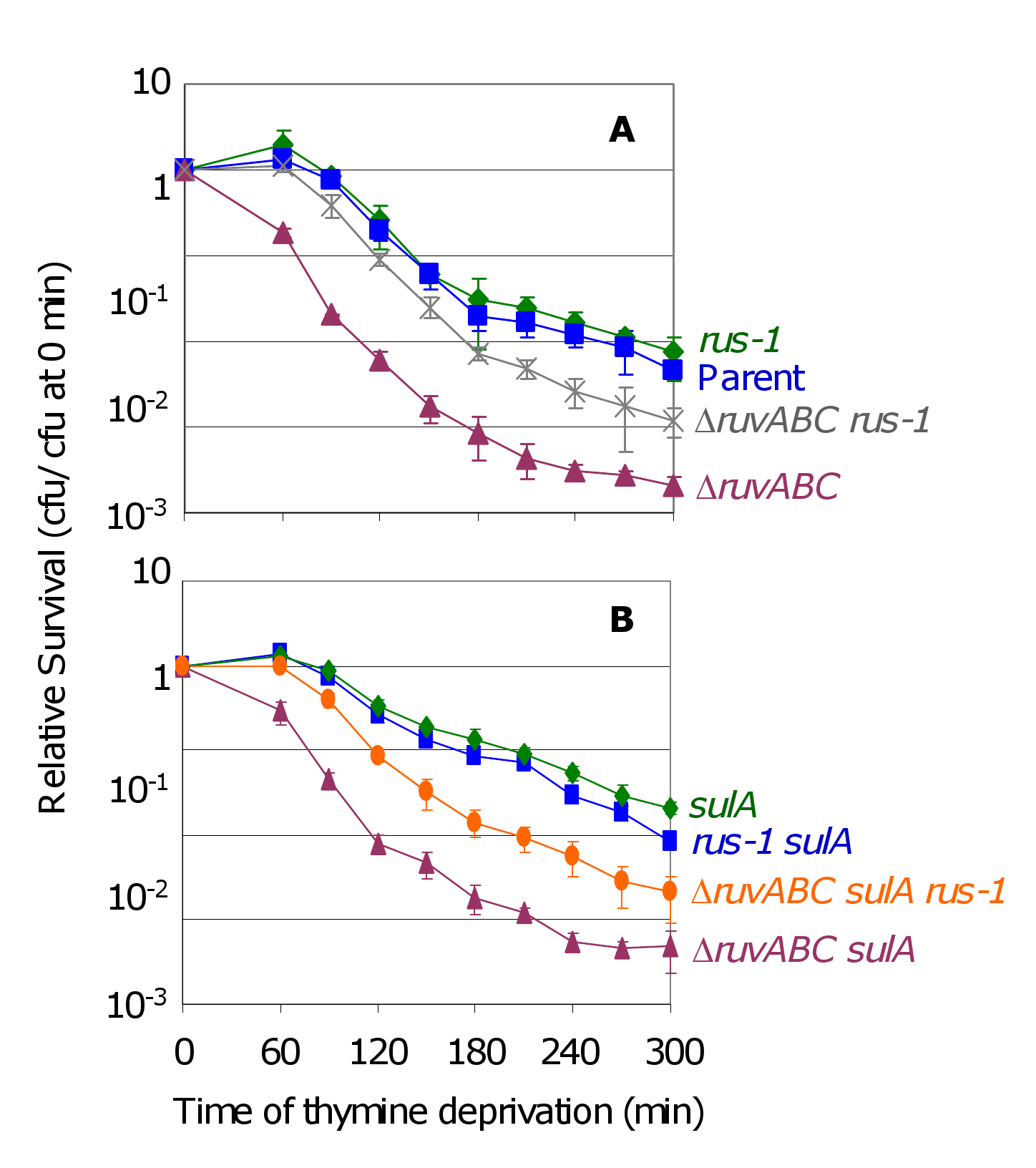

Supplement: Figure S5 — RusA expression partially reverses the hyper-TLD-sensitivity of ΔruvABC cells. (A) The RusA resolvase, expressed in rus-1 cells, partially restores TLD resistance to ΔruvABC cells. In the ΔruvABC (SMR10689, ▴) background, the rus-1 allele (SMR10690, ) increased resistance to TLD, but in the thy − rus + parental background (SMR10687, ▪) activating RusA via rus-1 mutation (SMR10686, ♦) did not have a significant effect. We cannot rule out the possibility that the lack of effect in Ruv+ cells is due to an inability of RusA to function when RuvABC are present (in vitro, RusA was inhibited by RuvA [McGlynn, et al]). Also, there is no reason to believe that rus-1 creates more resolution capacity than in wild-type cells, such that restoration to Ruv+ levels might be expected. (B) Possible RusA effects on TLD are not masked by SulA. Similar results to those in (A) are obtained even when RusA is activated in the absence of SulA. RusA activation partially suppressed the TLD hypersensitivity of ΔruvABC sulA cells (SMR10719, •, and SMR10718, ▴, respectively), but activating RusA in the absence of SulA (SMR10717, ▪) conferred no additional TLD-resistance over that conferred by sulA alone (SMR10716, ♦). This rules out the possibility that SulA expression might mask increased TLD-resistance of rus-1 cells by preventing cell division. Means ± SEM of 3 experiments (A,B). [McGlynn P, Lloyd RG, Marians KJ (2001) Formation of Holliday junctions by regression of nascent DNA in intermediates containing stalled replication forks: RecG stimulates regression even when the DNA is negatively supercoiled. Proc Natl Acad Sci U S A 98: 8235-8240.] (0.20 MB TIF) [file pgen.1000865.s005.tif]

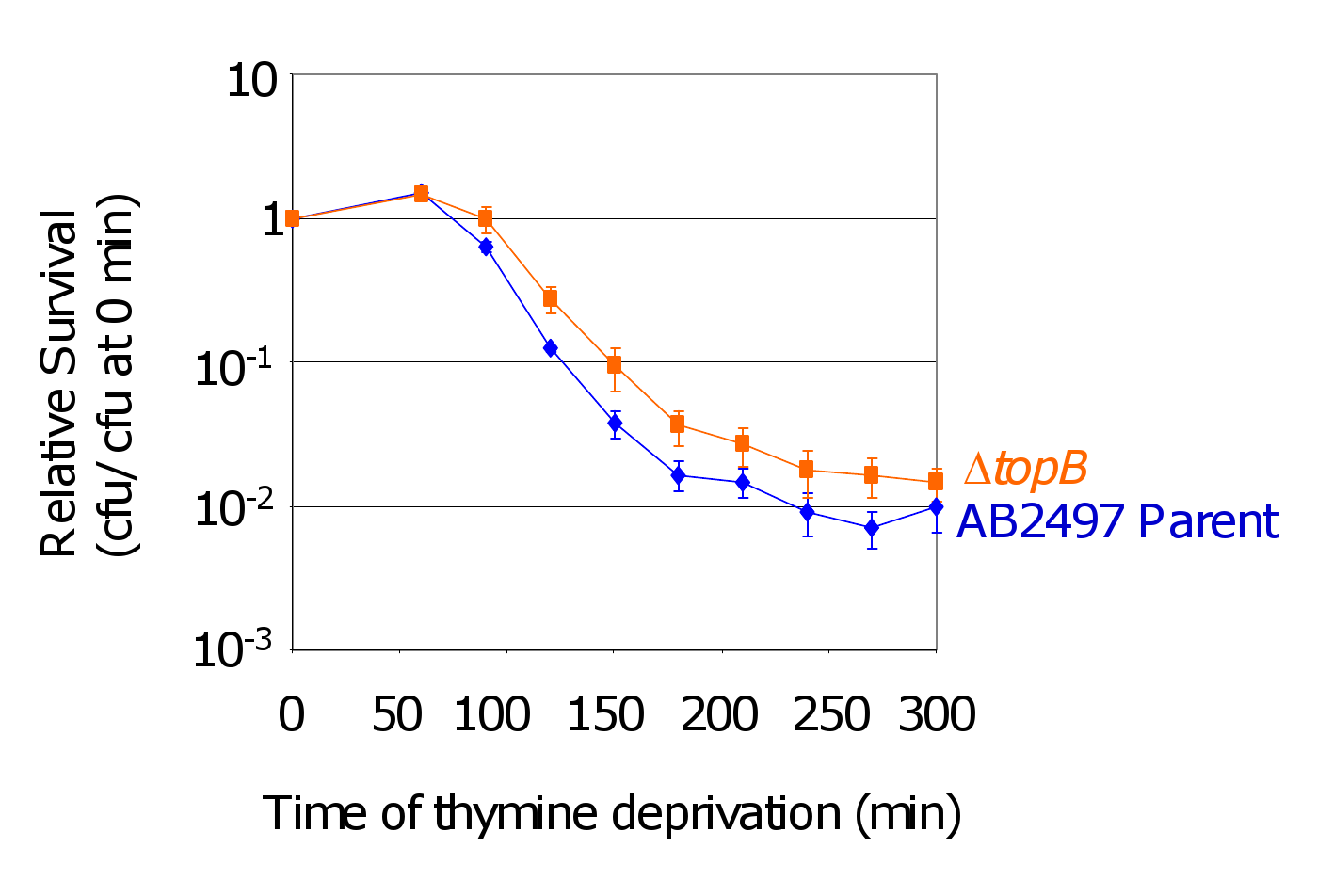

Supplement: Figure S6 — Topoisomerase III is not required for TLD. Cells lacking topB (SMR10672, ▪) are not significantly more resistant to TLD than their isogenic parental strain (AB2497; ♦), indicating that Topoisomerase III is not required for the RecQ-pathway of TLD in E. coli. Mean ± SEM of 3 experiments. (0.09 MB TIF) [file pgen.1000865.s006.tif]
