# Supplementary material for: Role of RecA and the SOS Response in Thymineless Death in Escherichia coli
Source: PLoS Genet. 2010 Mar 5;6(3):e1000865. doi: 10.1371/journal.pgen.1000865 (PMC2832678; doi:10.1371/journal.pgen.1000865)
Supplement: Table S1 — E. coli strains and plasmids used. (0.26 MB DOC) [file pgen.1000865.s007.doc]

**Table S1** *E. coli* strains and plasmids used

| Name | Genotype or Relevant Genotype | Reference or Source |
| --- | --- | --- |
| pCP20 | FLP recombinase vector | [1] |
| AB1157 | F^-^ *thi-1* *hisG4* Δ(*gpt-proA*)62 *argE3 thr-1 leuB6 araC14 lacY1 galK2 xylA5 mtl-1 rpsL31 tsx-33 glnV44 rfbC1 mgl-51 rpoS396 kdgK51* | CGSC1157 [2] |
| AB2497 | AB1157 *thyA12 deoB6* | CGSG2497 [3] |
| BW26355 | BW25113 Δ*recA635*::FRTKanFRT | CGSC7651 [1] |
| FC40 | Δ(*lac*-*proB*)_XIII_ *ara thi* Rif^R^ [F' *lacI*33Ω*lacZ proAB*^+^] | [4] |
| FC203 | FC40 *recA430* | [4] |
| GY8322 | AB1157 *sfiA11* Δ(*srlR-recA*)*306*::Tn*10* [mini-F K5353 *recA*^+^] | S. Sommers; ENZ280 [5] carrying the K5353 mini-F  plasmid [6] |
| HL353 | *thyA deo lac* | [7] |
| HL354 | HL353  *lexA3*(Ind^-^) | [7] |
| JC11450 | AB1157 Su^-^ | A. J. Clark |
| JJC754 | AB1157 Δ*ruvABC*::*cat* | [8] |
| JW0525 | Δ*intD*::FRTKanFRT | [9] |
| JW1752 | Δ*topB*::FRTKanFRT | [9] |
| JW2676 | Δ*srlR*::FRTKanFRT | [9] |
| JW2753 | Δ*chpA*(*mazF*)::FRTKanFRT | [9] |
| KL742 | LAM^-^ *thyA748*::Tn*10* *rph-1 deo-77* | CGSC6212 [10] |
| RDK1633 | *ruvB9* *zea3*::Tn*10* | R. Kolodner |
| RW117 | AB1157 *sulA100*::Tn*5* *pyrD* | R. Woodgate |
| SMR605 | AB1157 ruvB9 zea3::Tn10 | AB1157 x P1(RDK1633) |
| SMR818 | *recAo281* *srl300*::Tn*10* | [11] |
| SMR821 | *lexA3*(Ind^-^)  *malB::*Tn*9* | [12] |
| SMR1553 | FC40 *zea3*::Tn*10* | FC40 x P1(SMR605) |
| SMR1810 | FC40 *sulA100*::Tn*5* | FC40 x P1(RW117) |
| SMR3179 | FC40 *recJ*::Tn*10*Kan | [13] |
| SMR4314 | JC11450 *recJ*::Tn*10*Kan | JC11450 x P1(SMR3179) |
| SMR6201 | R594 Δ*recQ*::FRT*cat*FRT | [14] |
| SMR8097 | FC40 Δ*recF1804*::FRTKanFRT | [15] |
| SMR8987 | R594 Δ*recF1904*::FRT*cat*FRT | [16] |
| SMR10399 | AB1157 Δ*ruvABC*::*cat* *zea3*::Tn*10* | JJC754 x P1(SMR1553) |
| SMR10432 | KL724 Δ*recA635*::FRTKanFRT | KL742 x P1(BW26355) |
| SMR10433 | AB2497 Δ*recA635*::FRTKanFRT | AB2497 x P1(BW26355) |
| SMR10435 | KL742 Δ*recQ*::FRT*cat*FRT | KL742 x P1(SMR6201) |
| SMR10436 | AB2497 Δ*recQ*::FRT*cat*FRT | AB2497 x P1(SMR6201) |
| SMR10660 | AB2497 Δ*ruvABC*::*cat* *zea3*::Tn*10* | AB2497 x P1(SMR10399) |
| SMR10666 | FC40 *recA430* Δ*srlR*::FRTKanFRT | FC203 x P1(JW2676) |
| SMR10668 | AB2497 *recA430* Δ*srlR*::FRTKanFRT | AB2497 x P1(SMR10666) |
| SMR10669 | AB2497 *lexA3*(Ind^-^) *malB*::Tn*9* | AB2497 x P1(SMR821) |
| SMR10670 | AB2497 Δ(*srlR-recA*)*306*::Tn*10* | AB2497 x P1(GY8322) |
| SMR10672 | AB2497 Δ*topB*::FRTKanFRT | AB2497 x P1(JW1752) |
| SMR10673 | AB2497 *recAo281* *srlC300*::Tn*10* | AB2497 x P1(SMR818) |
| SMR10674 | AB2497 *sulA100*::Tn*5* | AB2497 x P1(SMR1810) |
| SMR10675 | HL353  *lexA3*(Ind^-^) *malB*::Tn*9* | HL353 x P1(SMR821) |
| SMR10676 | AB2497  *lexA3*(Ind^-^) *malB*::Tn*9* *recAo281* *srlC300*::Tn*10* | SMR10669 x P1(SMR818) |
| SMR10677 | AB2497 *sulA100*::Tn*5* Δ*recQ*::FRT*cat*FRT | SMR10674 x P1(SMR6201) |
| SMR10678 | *ruvA60 rus-1* Δ*intD*::FRTKanFRT | TNM759 x P1(JW0525) |
| SMR10681 | AB2497 Δ*recQ1906*::FRT | SMR10436 x pCP20 |
| SMR10683 | AB2497 Δ*recQ1906*::FRT  *lexA3*(Ind^-^) *malB*::Tn*9* | SMR10681 x P1(SMR821) |
| SMR10685 | AB2497 Δ*mazF*::FRTKanFRT | AB2497 x P1(JW2753) |
| SMR10686 | AB2497 *rus-1* Δ*intD*::FRTKanFRT | AB2497 x P1(SMR10678) |
| SMR10687 | AB2497 Δ*intD*::FRTKanFRT | AB2497 x P1(JW0525) |
| SMR10689 | AB2497 Δ*intD*::FRTKanFRT Δ*ruvABC*::*cat* *zea3*::Tn*10* | SMR10687 x P1(SMR10399) |
| SMR10690 | AB2497 *rus-1* Δ*intD*::FRTKanFRT Δ*ruvABC*::*cat* *zea3*::Tn*10* | SMR10686 x P1(SMR10399) |
| SMR10691 | AB2497 Δ*recF1804*::FRTKanFRT | AB2497 x P1(SMR8097) |
| SMR10692 | AB2497  *lexA3*(Ind^-^) *malB*::Tn*9* Δ*recF1804*::FRTKanFRT | SMR10669 x P1(SMR8097) |
| SMR10693 | AB2497 Δ*recF1904*::FRT*cat*FRT | AB2497 x P1(SMR8987) |
| SMR10694 | AB2497 *sulA100*::Tn*5* Δ*recF1904*::FRT*cat*FRT | SMR10674 x P1(SMR8987) |
| SMR10695 | AB2497 *recJ*::Tn*10*Kan | AB2497 x P1(SMR4314) |
| SMR10696 | AB2497  *lexA3*(Ind^-^)  *malB*::Tn*9* *recJ*::Tn*10*Kan | SMR10669 x P1(SMR4314) |
| SMR10709 | AB2497 Δ*intD*::FRT | SMR10687 x pCP20 |
| SMR10710 | AB2497 *rus-1* Δ*intD*::FRT | SMR10686 x pCP20 |
| SMR10711 | AB2497 Δ*intD*::FRT Δ*ruvABC*::*cat* *zea3*::Tn*10* | SMR10689 x pCP20 |
| SMR10712 | AB2497 *rus-1* Δ*intD*::FRT Δ*ruvABC*::*cat* *zea3*::Tn*10* | SMR10690 x pCP20 |
| SMR10713 | AB2497 *sulA100*::Tn*5* Δ(*srlR-recA*)*306*::Tn*10* | SMR10674 x P1(GY8322) |
| SMR10716 | AB2497 Δ*intD*::FRT *sulA100*::Tn*5* | SMR10709 x P1(SMR1810) |
| SMR10717 | AB2497 *rus-1* Δ*intD*::FRT *sulA100*::Tn*5* | SMR10710 x P1(SMR1810) |
| SMR10718 | AB2497 Δ*intD*::FRT Δ*ruvABC*::*cat* *zea3*::Tn*10 sulA100*::Tn*5* | SMR10711 x P1(SMR1810) |
| SMR10719 | AB2497 *rus-1* Δ*intD*::FRT Δ*ruvABC*::*cat* *zea3*::Tn*10 sulA100*::Tn*5* | SMR10712 x P1(SMR1810) |
| SMR10912 | AB2497  *lexA3*(Ind^-^)  *malB*::Tn*9* Δ*recA635*::FRTKanFRT | SMR10669 x P1(BW26355) |
| SMR10913 | AB2497 Δ*recQ1906*::FRT Δ*recA635*::FRTKanFRT | SMR10681 x P1(BW26355) |
| SMR11118 | AB2497 Δ*ruvABC*::*cat*  *zea3*::Tn*10* Δ*recA635*::FRTKanFRT | SMR10660 x P1(BW26355) |
| SMR11198 | AB2497 Δ*recQ1906*::FRT *recJ*::Tn*10*Kan | SMR10681 x P1(SMR4314) |
| SMR11205 | AB2497 Δ*recQ1906*::FRT Δ*recF1804*::FRTKanFRT | SMR10681 x P1(SMR8097) |
| TNM759 | AB1157 *rus-1* *ruvA60* | [17] |

CGSC, *E. coli* Genetic Stock Center, Yale.

Supplemental References

1. Datsenko KA, Wanner BL (2000) One-step inactivation of chromosomal genes in *Escherichia coli* K-12 using PCR products. Proc Natl Acad Sci U S A 97: 6640-6645.

2. Bachmann BJ (1972) Pedigrees of some mutant strains of *Escherichia coli* K-12. Bacteriol Rev 36: 525-557.

3. Howard-Flanders P, Boyce RP, Theriot L (1966) Three loci in *Escherichia coli* K-12 that control the excision of pyrimidine dimers and certain other mutagen products from DNA. Genetics 53: 1119-1136.

4. Cairns J, Foster PL (1991) Adaptive reversion of a frameshift mutation in *Escherichia coli*. Genetics 128: 695-701.

5. Dri AM, Rouviere-Yaniv J, Moreau PL (1991) Inhibition of cell division in hupA hupB mutant bacteria lacking HU protein. J Bacteriol 173: 2852-2863.

6. Dutreix M, Moreau PL, Bailone A, Galibert F, Battista JR, et al. (1989) New *recA* mutations that dissociate the various RecA protein activities in *Escherichia coli* provide evidence for an additional role for RecA protein in UV mutagenesis. J Bacteriol 171: 2415-2423.

7. Morganroth PA, Hanawalt PC (2006) Role of DNA replication and repair in thymineless death in *Escherichia coli*. J Bacteriol 188: 5286-5288.

8. Seigneur M, Bidnenko V, Ehrlich SD, Michel B (1998) RuvAB acts at arrested replication forks. Cell 95: 419-430.

9. Baba T, Ara T, Hasegawa M, Takai Y, Okumura Y, et al. (2006) Construction of *Escherichia coli* K-12 in-frame, single-gene knockout mutants: the Keio collection. Mol Syst Biol 2: 2006.0008.

10. Sat B, Reches M, Engelberg-Kulka H (2003) The *Escherichia coli mazEF* suicide module mediates thymineless death. J Bacteriol 185: 1803-1807.

11. Volkert MR, Margossian LJ, Clark AJ (1981) Evidence that *rnmB* is the operator of the *Escherichia coli recA* gene. Proc Natl Acad Sci U S A 78: 1786-1790.

12. McKenzie GJ, Harris RS, Lee PL, Rosenberg SM (2000) The SOS response regulates adaptive mutation. Proc Natl Acad Sci U S A 97: 6646-6651.

13. Harris RS (1997) In: Genetics Do, editor. On a Molecular Mechanism of Adaptive Mutation in *Escherichia coli*. Edmonton: University of Alberta. pp. 204.

14. Lopez CR, Yang S, Deibler RW, Ray SA, Pennington JM, et al. (2005) A role for topoisomerase III in a recombination pathway alternative to RuvABC. Mol Microbiol 58: 80-101.

15. Pennington JM, Rosenberg SM (2007) Spontaneous DNA breakage in single living *Escherichia coli* cells. Nat Genet 39: 797-802.

16. Magner DB, Blankschien MD, Lee JA, Pennington JM, Lupski JR, et al. (2007) RecQ promotes toxic recombination in cells lacking recombination intermediate-removal proteins. Mol Cell 26: 273-286.

17. Mandal TN, Mahdi AA, Sharples GJ, Lloyd RG (1993) Resolution of Holliday intermediates in recombination and DNA repair: indirect suppression of *ruvA*, *ruvB*, and *ruvC* mutations. J Bacteriol 175: 4325-4334.
